# Kawasaki disease in a northern region of Italy

**DOI:** 10.1186/1546-0096-9-S1-P81

**Published:** 2011-09-14

**Authors:** Elena Corinaldesi, MFabi, G Savorelli

**Affiliations:** 1 Department of Pediatry, Carpi, Italy; 2 Department of Pediatry, Bologna, Italy

## Objective

To examine the patient characteristics, diagnostic criteria, treatment, complications and outcomes of Kawasaki disease (KD) in a cohort of patients of two pediatric hospitals of Emilia Romagna from 2000 to 2010.

## Methods

Retrospective study: 26 patients diagnosed with complete or incomplete KD. Clinical and outpatient statuses were collected.

## Results

Male-to-female ratio was 1 to 1. Median age at onset: 35,8 months (2-101 months); children < 6 months: 23%. Complete KD: 73%. Age was not significant different between complete and incomplete KD.

Season of Onset of disease : winter in 35%, spring 31%, summer 11%, autumn 23%. Diagnostic signs: iperemic conjunctivitis and mucositis in 92% of pts, cutaneous rash in 88%, linfoadenopathy in 54%, alterations of extremities in 50%, desquamation of extremities in 58%; adjunctive signs: perineal dermatitis in 30% and diarrhea in 42%.

7 pts (5 boys) developed coronary involvement (group1): markers of inflammation and age at onset were not significantly different between group 1 and group 2 (without cardiac abnormalities). 28% of pts in group 1 was responder to therapy versus 47% in group 2. Coronary involvement regressed in all but 2 pts (both received late treatment).

## Conclusions

Male gender and late start of treatment were shown to be independent risk factors for developing coronary involvement, while younger age and incomplete form were not.

Late intravenous immunoglobulins seems to be risk factor for persistence of coronary aneurysm, but the small number of patients limits us to have definite conclusions .

